# Dataset on photodegradation of tetracycline antibiotic with zinc stannate nanoflower in aqueous solution – Application of response surface methodology

**DOI:** 10.1016/j.dib.2018.06.030

**Published:** 2018-06-22

**Authors:** Samira Taherkhani, Mohammad Darvishmotevalli, Kamaleddin Karimyan, Bijan Bina, Adibeh Fallahi, Hossein Karimi

**Affiliations:** a Department of Environmental Health Engineering, School of Public Health, Isfahan University of Medical Sciences, Isfahan, Iran; b Student Research Committee, Isfahan University of Medical Sciences, Isfahan, Iran; c Environmental Health Research Center, Kurdistan University of Medical Sciences, Sanandaj, Iran; d Department of Environmental Health Engineering, Faculty of Public Health, Tehran University of Medical Sciences, Tehran, Iran; e Student Research Committee, Kermanshah University of Medical Sciences, Kermanshah, Iran

**Keywords:** ZTO, Nanoflower, Photodegradation, Water treatment, Antibiotic, Modeling, RSM

## Abstract

Removal of pharmaceutical ingredients such as tetracycline from aqueous solution has a great importance. The aim of the current study was to investigate the degradation of tetracycline antibiotic in the presence of a triode semiconductor oxide as well as modeling of the photocatalytic degradation process in order to determine optimal condition Zinc stannate nanoflower (Zn_2_SnO_4_) was synthesized by hydrothermal process and characterized by X-ray diffraction (XRD), Fourier transform infrared (FT-IR), and scanning electron microscopy (SEM) techniques. Response surface methodology (RSM) was used to model and optimize four key independent variables, including photocatalyst dosage, initial concentration of tetracycline antibiotic (TC) as model pollutant, pH and reaction time of photocatalytic degradation. The proposed quadratic model was in accordance with the experimental results with a correlation coefficient of 98%. The obtained optimal experimental conditions for the photodegradation process were the following: zinc stannate (ZTO) dosage=300 mg L^-1^, initial concentration of TC= 10 mg L^-1^, reaction time= 100 min and pH=4.5. Under the optimal conditions, the predicted degradation efficiency was 95.45% determined by the proposed model. In order to evaluate the accuracy of the optimization procedure, the confirmatory experiment was carried out under the optimal conditions and the degradation efficiency of 93.54% was observed, which closely agreed with the predicted value.

**Specifications Table**

**Table:** 

Subject area	Environmental sciences
More specific subject area	Environmental chemistry
Type of data	Tables and figures
How data was acquired	In this study, Firstly, Zn_2_SnO_4_ was synthesized and investigated for TC removal in aqueous solution. After that, it characterized by XRD, FT-IR, and SEM techniques. Response surface methodology (RSM) was used to model and optimize four independent variables, including photocatalyst dosage, initial concentration of TC, pH and reaction time of photocatalytic degradation
Data format	Raw, analyzed
Experimental factors	Zinc stannate nanoflower (Zn_2_SnO_4_) was synthesized by hydrothermal process.
Experimental features	The samples preparation and analysis of them were performed according to standard method that provided invalid and similar references.
Data source location	Isfahan city, Iran
Data accessibility	Data are included in this article

**Value of the data**

• The treatment of wastewater containing TC by suitable and efficient ways (before entering the aquatic ecosystem) is very necessary. Based on this necessity, the data in this study provides information on the effectiveness of a new method for removal of TC from aqueous solutions.
• The obtained data showed the prepared ZTO has suitable efficiency for the removal of TC from aqueous solution. Accordingly, more research can be done with more hope and confidence on the present treatment method.
• The obtained data can be useful for future similar studies especially in terms of study design about removal survey of TC from aqueous solution.

## 1 Data

### 1.1 Modeling and optimization of the tetracycline degradation process during the (UV/ZTO) process via the response procedure method

The CCD method is used to design the experiments to achieve optimized conditions of tetracycline degradation. The designed experiments (31 experiments) are done on the proposed condition based on the CCD and the results are presented in Table 1. According to the data collected for determination of the degradation level, according to Table 1, a quadratic polynomial equation is obtained. The following equation shows a general model for prediction of the tetracycline degradation level according to real values:

$$Y=52.5714-30.6050\;\left(A\right)+5.4833\;\left(B\right)-3.5783\left(C\right)+20.8317\;\left(D\right)+8.0806\;\left(A^{2}\right)-7.0306\left(B^{2}\right)+1.8694\;\left(C^{2}\right)–9.1806\;\left(D^{2}\right)–13.1450\left(A\times B\right)–2.5450\left(A\times C\right)+1.3350\left(A\times D\right)–1.3550\left(B\times C\right)–0.1350\left(B\times D\right)+9.5650\left(C\times D\right)$$

**Fig. 1:**
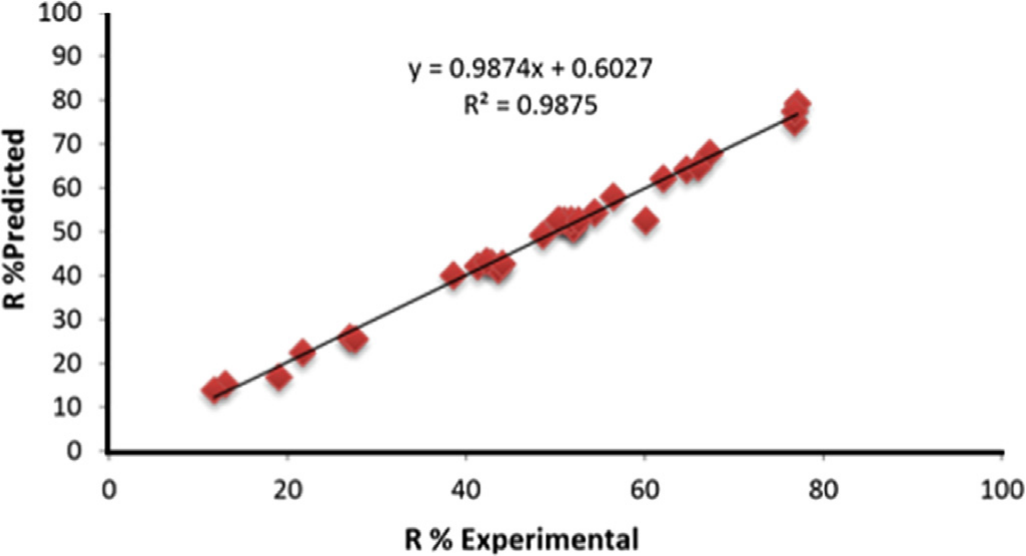
The relationship between the predicted and actual responses.

Where Y is the TC degradation degree, and A, B, C, and D are the real values of pH, photocatalyst dosage, initial concentration of TC, and reaction time. The predicted values of the tetracycline degradation are presented in Table 1 with a model. Drawing the predicted values with a model, according to the real values (Fig. 1), a line was achieved with the correlation coefficient of 0.98, which shows that the model is satisfactory.

**Table 1:** Experimental design matrix and the value of responses based on experiment run.

**Run**	**pH**	**ZTO dosage, mg/l**	**TC concentration, mg/l**	**Reaction time, min**	**Actual removal, %**	**Predicted removal, %**
1	6	150	25	40	42.8	42.91
2	9	250	25	40	13	15.25
3	7.5	200	20	70	51	52.57
4	7.5	200	20	130	64.7	64.22
5	7.5	300	20	70	52.1	51.02
6	6	150	15	40	48.6	49.32
7	6	250	25	100	77.12	79.24
8	7.5	200	30	70	52	50.86
9	9	250	25	100	43.6	41.16
10	9	150	15	40	27	25.9
11	9	200	20	70	11.8	13.88
12	6	250	15	40	62.1	62.13
13	4.5	200	20	70	76.8	75.09
14	9	150	25	40	19	16.94
15	6	250	25	40	54.4	54.36
16	7.5	100	20	70	38.6	40.01
17	9	250	15	100	41.3	21.42
18	7.5	200	20	10	21.7	22.55
19	7.5	200	20	70	51.8	22.55
20	9	150	25	100	42.3	43.29
21	6	150	25	100	67.3	67.93
22	7.5	200	20	70	50.5	52.57
23	9	250	15	40	27.6	25.5
24	9	150	15	100	44.06	42.68
25	7.5	200	20	70	51.7	52.57
26	7.5	200	20	70	50.3	52.57
27	7.5	200	10	70	56.5	58.01
28	6	200	15	100	76.8	77.44
29	7.5	200	20	70	60.1	52.57
30	6	150	15	100	66	64.77
31	7.5	200	20	70	52.6	52.57

The results obtained from the ANOVA, which are driven from the Mini Tab software, are presented in Table 2.

**Table 2:** Analysis of variance (ANOVA) for the selected quadratic model.

Source	DOF	Adj SS	Adj MS	F-value	P-value
Regression	14	9079.61	648.54	89.96	0.000
Residual	16	115.34	7.21	–	–
Total	30	9194.96	–	–	–

SS: Sum of squares.

MS: Mean squares.

P values related to the terms of the proposed model for the TC degradation process during the UV/ZTO process are presented in Table 3.

**Table 3:** The ANOVA results for the coefficients of variables of quadratic model.

**Factor**	**Coefficient**	**P-Value**
A	−30.605	0.000
B	5.4883	0.000
C	−3.5783	0.005
D	20.8317	0.000
A^2^	−8.0806	0.001
B^2^	−7.0306	0.001
C^2^	1.8694	0.336
D^2^	−9.1806	0.000
A×B	−13.145	0.000
A×C	−2.545	0.000
A×D	1.335	0.626
B×C	−1.355	0.621
B×D	−0.135	0.961
C×D	9.565	0.003

The optimized values of the chosen variables and the maximum predicted value for the tetracycline degradation are presented in Table 4. To evaluate the validity of the predicted value, the experimental would be done via CCD in the same proposed condition and with a value of 95.45% for the TC degradation in the optimized conditions.

**Table 4:** Optimized values of parameters effective on the tetracycline degradation.

**Parameters**	**Optimized amounts**
ZTO (mg/L)	300
pH	4.5
mg/L) )TC	10
Time(min)	100
Removal Percent	93.54

### 1.2 Evaluation of synthesized nano-particles properties

**Fig. 2:**
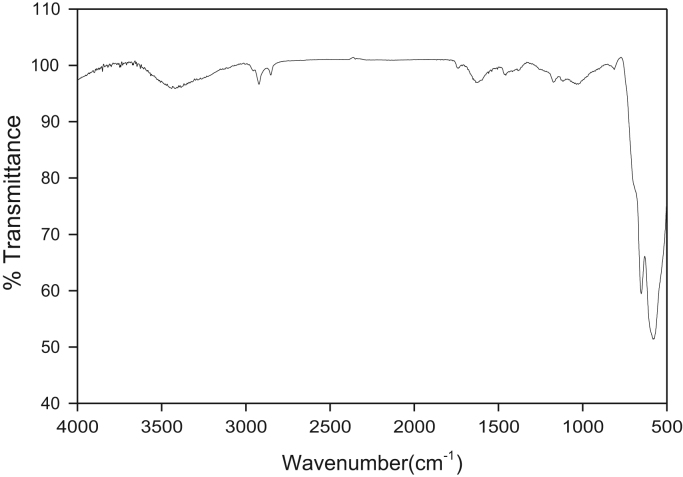
FT-IR spectrum of prepared ZTO.

FT-IR studies on the synthesized ZTO via the 500–4000 cm^−1^ hydrothermal method are evaluated and the result is shown in Fig. 2.

**Fig. 3:**
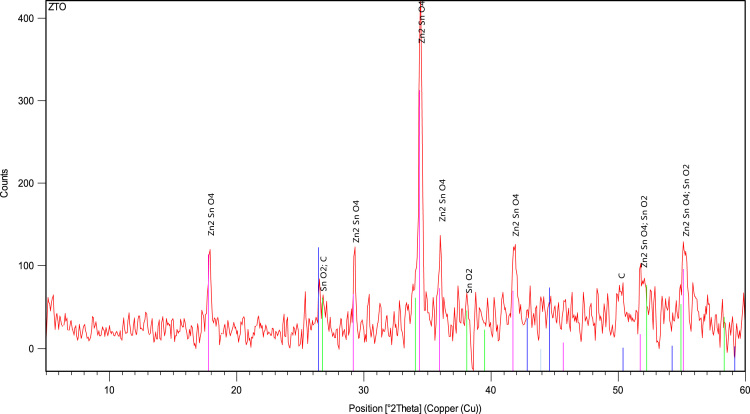
XRD pattern spectrum of prepared ZTO.

Position and relative intensity of peaks in the XRD pattern of the synthesized ZTO indicates the presence of crystal phases (with the cart No. of 2184-074-01) in the structure of the synthesized photocatalyst (Fig. 3).

**Fig. 4:**
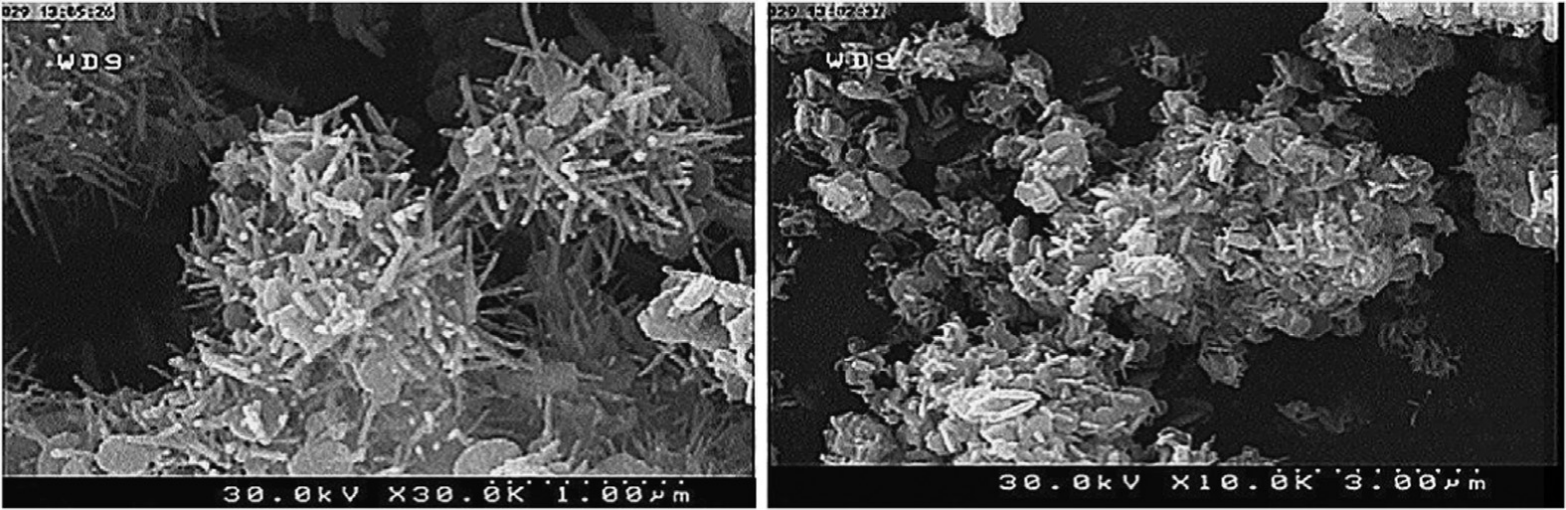
SME images of prepared ZTO.

The SEM images of the synthesized ZTO via the hydrothermal method are presented in Fig. 4. It was observed that the ZTO is in the form of nano flowers.

### 1.3 The effect of different parameters on the photocatalytic degradation of TC

#### 1.3.1 Effect of initial concentration of pollutant and contact time on the tetracycline degradation

**Fig. 5:**
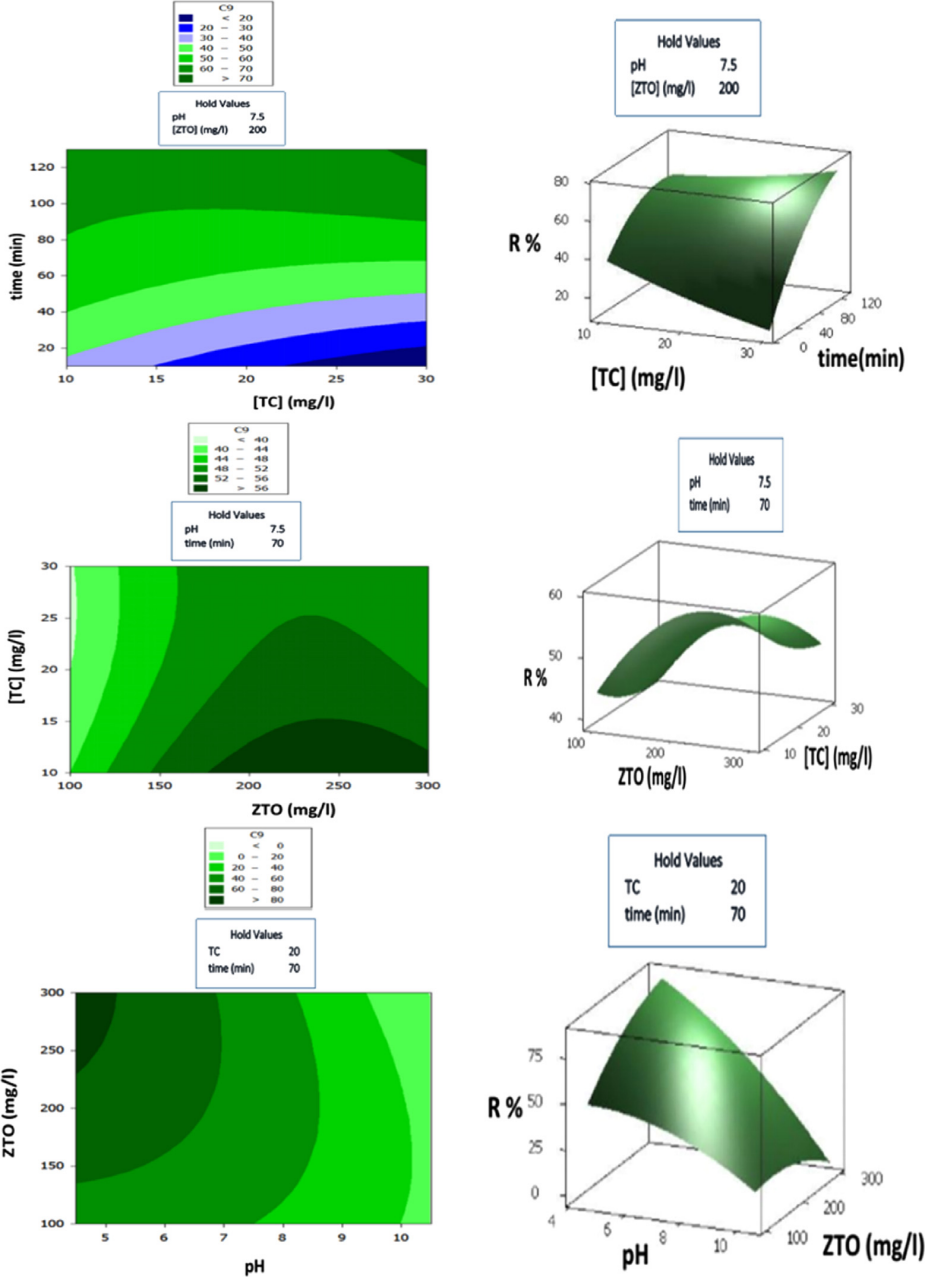
Surface and counter plots of the photocatalytic degradation of tetracycline.

Fig. 5-a shows the effect of initial concentration of pollutant and contact time on the tetracycline degradation (pH is 7.5 and the photocatalyst dosage is 200 mg L^−1^). The tetracycline degradation efficiency increases with an increase in contact time and the pollutant concentration.

#### 1.3.2 Effect of initial concentration of pollutant and photocatalyst dosage on the tetracycline degradation

**Fig. 6:**
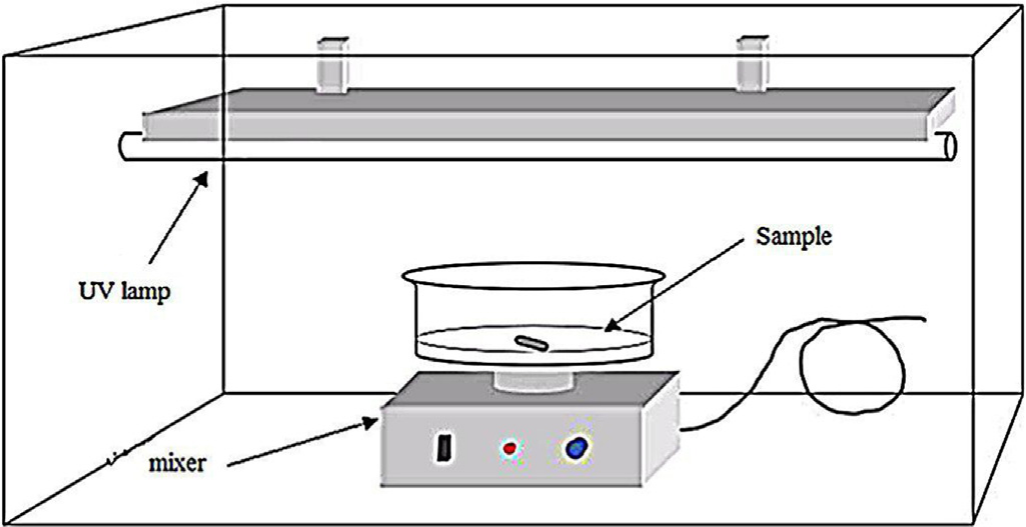
The schematic of UV photoreactor.

The tetracycline degradation degree for the reaction time of 70 min and pH of 7.5, as a function of photocatalyst dosage, is shown in Fig. 6-b. The obtained results from the diagram indicate that in the low concentration of pollutant, the degradation degree increases as a result of the existence of numerous absorption sites.

#### 1.3.3 Effects of pH and initial concentration of pollutant on tetracycline degradation

In Fig. 5-b in the conditions that the time is equal to 70 min and pollutant concentration is 20 mg L^−1^ in the acidic medium the highest amount of degradation occurs as a result of the electrostatic attraction between the pollutant and the photocatalyst surface.

## 2 Experimental design, materials and methods

### 2.1 Properties of tetracycline antibiotic

The properties of the tetracycline antibiotic as pollutant sample are shown in Table 5.

**Table 5:** The properties of TCA.

**Parameters**	**Properties**
Molecular formula	C_22_H_24_O_8_N_2_HCl
Molecular weight (g/mol)	480.9
Solubility (mol/L)	0.041
λ _max_ (nm)	359
	
Chemical structure	

### 2.2 Materials

The materials used in this investigation are tetracycline antibiotic (C_22_H_24_O_8_N_2_HCl), tin tetrachloride (pentahydrate) 98%, hexahydrate zinc nitrate, 98% (Sigma-Aldrich Co.), sodium hydroxide, ammonia, 32%, ethanol (Merck Co.).

The used equipment includes the following: the digital pH meter (Metrohm 780/Swiss) was used to adjust the pH of the solution, the spectrophotometer (Shimadzu UV-160/japan), magnetic stirrer (Helidolph Mr 3001, k/Germany), ultrasonic bath (CD-4820), autoclave, digital oven (Pars Azma), electronic furnace (Syborn Thermolyne, 1500 Furnace) with precision of +0.00001.

### 2.3 Synthesis of Zn_2_SnO_4_

The following steps were taken to synthesize Zn_2_SnO_4_:1.5 mg of SnCl_4_.5H_2_O and 3 mg of Zn (NO_3_)_2_.6H_2_O were separately dissolved in 20 ml of double distilled water. Then, 20 ml of sodium hydroxide (1 M) was added drop by drop to the stirring solution of SnCl_4_.5H_2_O. Finally, the zinc nitrate solution was added to the above solution to caused formation of white dye hybrid sediment. The obtained sediment was transported to Teflon autoclave (200–220 °C) for 48 h. At the end, the sediment was filtered and washed with water and ethanol, then was dried in oven at 80 °C for 20 h [1], [2].

### 2.4 Evaluation of the photocatalytic destruction of the synthesized nanoparticle

A Photocatalytic activity of the synthesized ZTO for destruction of the TC was evaluated under irradiation of UV light (30 W) (UV-C). In order to carry out the experiment, 100 ml of the solution of the pollutant was poured in 200 ml Bécher as a reactor with magnetic stirrer (Fig. 6).

In order to determine the concentration of pollutant at any time, the sampling accrued in intervals of 0–100 min and the absorption of antibiotic solution was recorded with the spectrophotometer in the wavelength of 359 nm. The removal degree was calculated using the following equation [3], [4], [5], [6], [7], [8], [9].

$$\mathrm{Removal},\%=\left[\left(C_{0}-C_{t}\right)/C_{0}\right]\times 100$$

Where C_0_ is initial concentration of TC and C_t_ is the concentration of TC at time t.

### 2.5 Optimizing the photocatalytic degradation process

To optimize the process of the photocatalytic degradation, central composite design (CCD) was used- RSM׳s common form [10], [11], [12], [13], [14]. Considering the initial experiments, the four factors of pH, initial density, photocatalyst dosage and reaction time, were investigated as the main effective factors and the antibiotic degradation degree was considered as the response. Table 6 shows Levels of independent variables for photocatalytic degradation of TC. The intended design, presented in Table 7 is based on CCD and considers the four variable including 31 experiments with various conditions.

**Table 6:** Factors and levels of designing experiments via the CCD method.

**Parameters**	**Level**				
	**-2**	**1-**	**0**	**1+**	**2+**

					
(X_1_) pH	4.5	6	7.5	9	10.5
(X_2_) ZTO	100	150	200	250	300
(X_3_) TC	10	15	20	25	30
(X_4_) Time	10	40	70	100	130

**Table 7:** Designing of experiment via the CCD method based on the real values of the variables.

**Run**	**X**_**1**_	**X**_**2**_**(mg L**^**−1**^**)**	**X**_**3**_**(mg L**^**−1**^**)**	**X**_**4**_**(min)**
1	6	150	25	40
2	9	250	25	40
3	7.5	200	20	70
4	7.5	200	20	130
5	7.5	300	20	70
6	6	150	15	40
7	6	250	25	100
8	7.5	200	30	70
9	9	250	25	100
10	9	150	15	40
11	10.5	200	20	70
12	6	250	15	40
13	4.5	200	25	70
14	9	150	25	40
15	6	250	20	40
16	7.5	100	15	70
17	9	250	20	100
18	7.5	200	20	10
19	7.5	200	25	70
20	9	150	25	100
21	6	150	25	100
22	7.5	200	25	70
23	9	250	25	40
24	9	150	15	100
25	7.5	200	20	70
26	7.5	200	20	70
27	7.5	200	10	70
28	6	200	15	100
29	7.5	200	20	70
30	6	150	15	100
31	7.5	200	20	70

These experiments include 16 factorial experiment at factor levels of -1 and +1, seven experiments at central levels (0), and eight experiments at axial points (α=2). To create connection between independent and dependent variables (presenting a model, introducing the process) the following Quadratic polynomial equation is used [15], [16], [17], [18], [19], [20].

$$y=b_{o}+\sum_{i=1}^{n}(b_{i}x_{i})+\sum_{i=1}^{n}(b_{ii}x_{ii}^{2})+\sum_{i,j=1}^{n}\left(b_{ij}x_{i}x_{j}\right)$$

Where, y is the response predicted by the model, x_i_ is the encoded amount of levels of variables and b_o_, b_i_, b_ii_, and b_ij_ are the coefficients of the model.
